# Cost of Inpatient Falls and Cost-Benefit Analysis of Implementation of an Evidence-Based Fall Prevention Program

**DOI:** 10.1001/jamahealthforum.2022.5125

**Published:** 2023-01-20

**Authors:** Patricia C. Dykes, Mica Curtin-Bowen, Stuart Lipsitz, Calvin Franz, Jason Adelman, Lesley Adkison, Michael Bogaisky, Diane Carroll, Eileen Carter, Lisa Herlihy, Mary Ellen Lindros, Virginia Ryan, Maureen Scanlan, Mary-Ann Walsh, Matthew Wien, David W. Bates

**Affiliations:** 1 Center for Patient Safety, Research, and Practice, Department of General Internal Medicine and Primary Care, Brigham and Women’s Hospital, Boston, Massachusetts; 2 Harvard Medical School, Boston, Massachusetts; 3 Eastern Research Group, Lexington, Massachusetts; 4 Division of General Medicine, Columbia University Irving Medical Center/NewYork-Presbyterian, New York, New York; 5 Department of Nursing, Newton Wellesley Hospital, Newton, Massachusetts; 6 Division of Geriatrics, Montefiore Medical Center, Bronx, New York; 7 Munn Center for Nursing Research, Department of Nursing and Patient Care Services, Massachusetts General Hospital, Boston, Massachusetts; 8 School of Nursing, Columbia University, New York, New York; 9 Department of Patient Safety and Quality, North Shore Medical Center, Salem, Massachusetts; 10 Department of Nursing and Patient Care Services, Montefiore Medical Center Hospitals, Bronx, New York; 11 Department of Nursing and Patient Care Services, Brigham and Women’s Faulkner Hospital, Boston, Massachusetts

## Abstract

**Importance:**

There is insufficient research on the costs of patient falls in health care systems, a leading source of nonreimbursable adverse events.

**Objective:**

To report the costs of inpatient falls and the cost savings associated with implementation of an evidence-based fall prevention program.

**Design, Setting, and Participants:**

In this economic evaluation, a matched case-control study used the findings from an interrupted time series analysis that assessed changes in fall rates following implementation of an evidence-based fall prevention program to understand the cost of inpatient falls. An economic analysis was then performed to assess the cost benefits associated with program implementation across 2 US health care systems from June 1, 2013, to August 31, 2019, in New York, New York, and Boston, Massachusetts. All adults hospitalized in participating units were included in the analysis. Data analysis was performed from October 2021 to November 2022.

**Interventions:**

Evidence-based fall prevention program implemented in 33 medical and surgical units in 8 hospitals.

**Main Outcomes and Measures:**

Primary outcome was cost of inpatient falls. Secondary outcome was the costs and cost savings associated with the evidence-based fall prevention program.

**Results:**

A total of 10 176 patients who had a fall event (injurious or noninjurious) with 29 161 matched controls (no fall event) were included in the case-control study and the economic analysis (51.9% were 65-74 years of age, 67.1% were White, and 53.6% were male). Before the intervention, there were 2503 falls and 900 injuries; after the intervention, there were 2078 falls and 758 injuries. Based on a 19% reduction in falls and 20% reduction in injurious falls from the beginning to the end of the postintervention period, the economic analysis demonstrated that noninjurious and injurious falls were associated with cost increases of $35 365 and $36 776, respectively. The implementation of the evidence-based fall prevention program was associated with $14 600 in net avoided costs per 1000 patient-days.

**Conclusions and Relevance:**

This economic evaluation found that fall-related adverse events represented a clinical and financial burden to health care systems and that the current Medicare policy limits reimbursement. In this study, costs of falls only differed marginally by injury level. Policies that incentivize organizations to implement evidence-based strategies that reduce the incidence of all falls may be effective in reducing both harm and costs.

## Key Points

**Question:**

What are the costs of inpatient falls and cost benefits associated with the Fall TIPS (Tailoring Interventions for Patient Safety) Program?

**Findings:**

In this economic evaluation using a large cohort (900 635 patients; 7858 noninjurious falls; 2317 injurious falls), the average total cost of a fall was $62 521 ($35 365 direct costs), and injury was not significantly associated with increased costs. The Fall TIPS Program was associated with $22 million in savings at study sites across the 5-year study period.

**Meaning:**

The findings of this study indicate that implementation of cost-effective, evidence-based safety programs was associated with lower cost and care burdens associated with inpatient falls and are a step toward safer, more affordable patient care.

## Teaser

This economic evaluation reports on the costs of inpatient falls and the cost savings associated with implementation of an evidence-based fall prevention program.

## Introduction

Preventable medical errors and adverse events in US hospitals are well documented,^1,2,3,4^ and the estimated costs total $17 billion annually.^3,5,6^ Falls comprise the largest category of preventable adverse events in hospitals,^7,8,9^ and the associated per-patient costs are estimated to range from $351 to $13 616,^10,11,12^ but research validating costs is needed.

Many hospital falls can be prevented through implementation of an evidence-based program that identifies each patient’s fall risk factors, develops individualized prevention plans, and consistently implements the plans through staff and patient engagement.^8,13,14^ However, adoption of such programs is limited. The purpose of this study was to use electronic health record (EHR) data to estimate the cost of falls and related injuries and to analyze the costs and benefits associated with implementing the Fall TIPS (Tailoring Interventions for Patient Safety) Program, an evidence-based, freely available fall prevention program associated with a 15% to 25% reduction in inpatient falls and a 0% to 34% reduction in injurious falls.^8,15^

We assessed the costs of inpatient falls (2021 US dollars) before, during, and after implementation of the Fall TIPS Program across 2 large health care systems. We categorized fall severity on a scale from noninjurious to severe or death^16^ to understand how degrees of injury are associated with costs. Finally, we assessed the cost benefits associated with program implementation.

## Methods

### Study Design and Participants

In this economic evaluation, we performed a cost-benefit analysis of implementing the Fall TIPS Program with the primary outcome of cost of inpatient falls. A secondary analysis quantified the costs and savings associated with the evidence-based fall prevention program. Our base model estimated the total cost savings of intervention effects (ie, reduction in overall direct and total costs of hospital stay). The model was framed from the perspective of the health care system, and data on costs and outcomes were obtained from a nonrandomized interrupted time series (ITS) study conducted across 2 large health care systems in the Bronx, New York (site 1; 3 hospitals), and Boston, Massachusetts (site 2; 5 hospitals). All hospitals implemented the Fall TIPS Program on medical and surgical units. The ITS evaluation was conducted between June 1, 2013, and August 31, 2019, to evaluate the Fall TIPS Program’s outcomes and compared the falls and fall injury rates (eAppendix and eFigures 1 and 2 in Supplement 1). We also conducted a case-control study to estimate the additional direct and total costs associated with fall and injury level. This study followed the Consolidated Health Economic Evaluation Reporting Standards (CHEERS) and Strengthening the Reporting of Observational Studies in Epidemiology (STROBE) reporting guidelines. The institutional review boards for Montefiore and Mass General Brigham Healthcare Systems approved the study. Due to the quality-improvement nature of the intervention, a waiver of informed consent was granted by the institutional review boards of Montefiore and Mass General Brigham Healthcare Systems.

#### Study Design and Intervention

All sites had a 15-month preintervention period from June 2013 to September 2014. In collaboration with hospital leadership, the study team assigned the go-live period between September 2014 and May 2018 based on EHR go-live dates and other competing projects (Figure). All hospitals implemented the Fall TIPS risk assessment and care planning tools in their EHR. Boston sites also implemented the Fall TIPS laminated poster.^8,14^ New York sites implemented the EHR-generated Fall TIPS poster.^8,14^ Both modalities are effective in facilitating patient engagement in fall prevention.^17^ Protocol adherence was measured with patient engagement audits conducted by unit-based champions.^8^ All sites completed data collection in August 2019.

**Figure.**
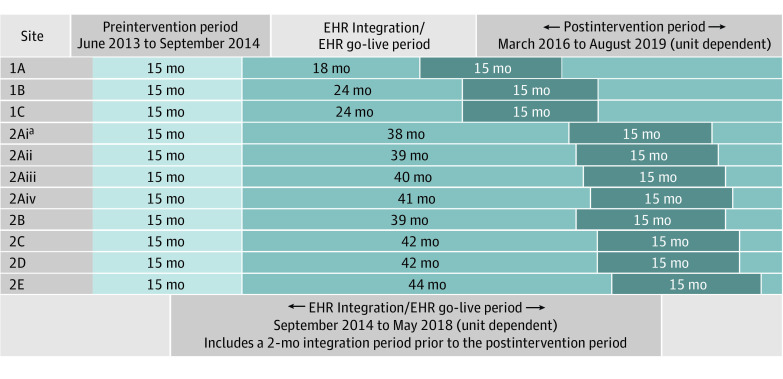
Preintervention, Electronic Health Record (EHR) Integration/EHR Go-Live, and Postimplementation Periods by Site ^a^Site 2A (consisting of 26 units within 1 hospital) staggered the postintervention period. All other hospital sites used the same start and end dates for all units. Roman numerals indicate the grouping of when each unit started the postintervention period.

#### Outcomes

The primary outcomes were the total and direct costs of falling compared with not falling. The secondary outcomes were the costs and cost savings associated with the evidence-based fall prevention program.

### Statistical Analysis

#### Case-Control Study

We assessed the costs of falling compared with not falling. Cases were defined as all patients who were reported as falling in the incident reporting system of participating hospitals from June 1, 2013, through August 31, 2019. Controls were randomly selected patients who were not reported as falling during the same periods. Controls were matched to cases in a 3:1 ratio based on unit, sex, race and ethnicity, insurance type, Charlson Comorbidity Index score, age, and pre-event length of stay (LOS). Resource use data were obtained from the financial database and comprised the total and direct costs. Total costs include administrative and overhead costs. Because total costs cannot be avoided in the short term, they may overestimate cost savings. Indirect costs can be adjusted over time; fewer falls will require less administrative time, lowering indirect costs. Resource use indicators included LOS and costs. We first evaluated the associations of falls with no injury, any injury, minor injury, and severe injury with log of cost, a continuous dependent variable (using the log transformation because cost was right skewed), using random-effects models to account for matching. We then reexamined these associations using random-effects regression to control for potential confounding due to clinical and socioeconomic factors. Data analysis was performed from October 2021 to November 2022. We used SAS statistical software, version 9.4 (SAS Institute) for the analyses.

#### Cost-Benefit Analysis

We leveraged the findings of the case-control study to determine costs and benefits associated with implementing the Fall TIPS Program.

##### Costs of Implementation

One-time noncapital costs and the ongoing annual operational costs associated with implementation were obtained from study sites. The program was integrated into EHR systems, and there were no license fees or capital costs. One-time implementation costs included personnel training (ie, 1 hour per nurse/2 hours per nurse champion), laminated posters, dry erase markers, adhesives (Boston hospitals), and poster replacement costs (20% of posters per year, amortized over 5 years); Boston hospitals spend $3.33 per bed per year. Bronx hospitals print new paper posters for each patient at an estimated cost of $1.00 per patient. Ongoing costs may be incurred because of additional time spent using the Fall TIPS Program. A survey indicated a majority of nurses (62%) averaged 1-minute additional time each day per patient, while 38% reported averaging a 10-minute time savings each day per patient.^18^ Because of the broad range of results, we assume that the Fall TIPS Program is time neutral.

##### Benefits of Implementation

The benefits attributable to the Fall TIPS Program included any reduction in patient falls and injurious falls after implementation. We value the benefits as the avoided costs of evaluating and treating a patient who had a fall event. We obtained figures for the average cost of a hospital stay for a patient with (1) no fall, (2) a fall but no injury, and (3) a fall with any injury. This narrowly focused estimate of benefits does not include the pain and suffering avoided by the patient, nor the opportunity cost of the patient’s time had they required a longer LOS.

##### Cost-Benefit Analysis

We compared the costs and benefits of the Fall TIPS Program to evaluate a snapshot of the costs and benefits at the start and at the end of the intervention periods.

## Results

### Study Design and Participants

A total of 900 635 patients and 4 955 534 patient-days were included in the ITS (Table 1; eAppendix and eFigures 1 and 2 in Supplement 1). Across periods, patients were similar in hospital and unit LOS, age, sex, and insurance type. Patients at site 1 were more likely to be non-White than White (79.8%-82.6% non-White [American Indian or Alaska Native, Asian or Pacific Islander, Black or African American, Native Hawaiian or Other Pacific Islander] across periods), whereas patients at site 2 were more likely to be White than non-White (82.1%-83.3% White across periods). The mean (SD) hospital LOS ranged from 5.4 (7.3) days to 5.6 (7.9) days, and the mean (SD) unit LOS ranged from 4.4 (6.1) days to 4.6 (6.0) days. There were more women in each period than men (52.3%-53.6% women vs 46.5%-47.7% men). More patients were younger than 65 years in all periods (51.1%-56.7% patients were <65 years; 43.3%-48.9% patients were ≥65 years). Standardized differences comparing demographic characteristics across periods were well balanced (<10%), except for race and ethnicity when comparing the preintervention period with the go-live period and the total Charlson Comorbidity Index score at admission when comparing the preintervention period with the postintervention period. We adjusted for these variables in the analyses (see eTables 1 and 2 in Supplement 1 for site-specific demographic characteristics).

**Table 1.** Patient Demographic Characteristics (Sites 1 and 2)

Characteristic	Patients, No. (%)			Standardized difference, %^a^		
	Before the intervention/EHR go-live period	EHR integration/EHR go-live period	Postintervention period	Before EHR integration/EHR go-live period	EHR integration/EHR go-live period	Postintervention period
No. of admissions	195 528	489 597	215 510	NA	NA	NA
No. of patient-days	1 102 437.52	2 641 765.57	1 211 330.63	NA	NA	NA
Hospital length of stay, mean (SD), d	5.6 (7.9)	5.4 (7.3)	5.6 (7.5)	3.2	0.2	−3.0
Unit length of stay, mean (SD), d	4.6 (6.0)	4.4 (6.1)	4.4 (5.7)	3.6	3.1	−0.7
Age, y						
<65	110 800 (56.7)	259 723 (53.1)	110 059 (51.1)	7.3	4.0	1.2
≥65	84 728 (43.3)	229 867 (47.0)	105 451 (48.9)	−7.3	−4.0	−1.2
Race^b^						
Non-White	66 528 (35.2)	140 688 (29.5)	73 306 (35.3)	12.2	−12.4	−0.2
White	122 187 (64.8)	335 723 (70.5)	134 132 (64.7)	−12.2	12.4	0.2
Ethnicity^c^						
Hispanic	31 365 (18.9)	61 491 (13.8)	34 714 (17.4)	13.8	−9.7	4.1
Non-Hispanic	134 231 (81.1)	382 795 (86.2)	165 113 (82.6)	−13.8	9.7	−4.1
Sex						
Female	104 700 (53.6)	255 946 (52.3)	113 406 (52.6)	2.6	−0.7	1.9
Male	90 816 (46.4)	233 638 (47.7)	102 100 (47.4)	−2.6	0.7	−1.9
Primary insurance						
Public	121 053 (62.7)	293 914 (60.3)	134 363 (62.5)	4.9	−4.6	0.4
Private	72 133 (37.3)	193 765 (39.7)	80 650 (37.5)	−4.9	4.6	−0.4
Total Charlson Comorbidity Index score at admission						
0-1	92 551 (48.6)	228 221 (46.6)	92 662 (43.0)	3.8	7.3	11.2
≥2	98 087 (51.4)	261 175 (53.4)	122 817 (57.0)	−3.8	−7.3	−11.2

Abbreviations: EHR, electronic health record; NA, not applicable.

^a^ Considering standard differences of less than 10% as not significant.

^b^ Race data are based on self-reported EHR data. Racial and ethnic categories in the non-White group include American Indian or Alaska Native, Asian or Pacific Islander, Black or African American, Native Hawaiian or Other Pacific Islander. Categories in the White group include White or Caucasian.

^c^ Ten percent of ethnicity data missing across sites.

### Case-Control Study

During the 74-month study period, there were 7858 noninjurious and 2317 injurious falls. Table 2 includes the descriptive statistics. The average total cost of a fall was $62 521 ($36 776 direct costs), and the average total cost of a fall with any injury was $64 526 (Table 3). The intervention cost $267 700 (both health care systems), equivalent to $0.88 per patient or $180 per 1000 patient-days. The intervention prevented 567 falls (142 with injury and 425 without injury), resulting in avoided total costs per 1000 patient-days of $14 762 (approximately $8500 in direct costs per 1000 patient-days) (Table 4) in the postintervention period. The net avoided costs per 1000 patient-days totaled $14 600 (approximately $8300 direct costs) for total cost savings of $22 036 714.

**Table 2.** Descriptive Statistics of Case and Control Groups

Characteristic	Patients, %		Standardized difference, %
	Cases	Controls	
No. of admissions	10 176	29 161	NA
No. of patient-days	126 374	119 020	NA
Hospital length of stay, mean (SD), d	15.0 (18.5)	6.9 (8.2)	NA
Unit length of stay, mean (SD), d	12.4 (16.3)	5.3 (6.2)	NA
Age, y			
<65	47.3	49.0	2.8
≥65	52.7	51.0	
Race and ethnicity			
Non-White	33.1	32.7	0.7
White	66.9	67.3	
Sex			
Female	46.6	46.2	0.7
Male	53.4	53.8	
Primary insurance			
Public	69.4	69.5	0.3
Private	30.6	30.5	
Total Charlson Comorbidity Index score at admission			
0-1	28.1	31.2	5.6
≥2	71.9	68.8	

Abbreviation: NA, not applicable.

**Table 3.** Cost Table

Category of fall injury	Cases (falls)		Matched controls		Difference, mean (95% CI), $
	No.	Mean (95% CI), $	No.	Mean (95% CI), $	
Fall, no injury					
Average direct cost per patient	7858	35 365 (30 515-40 216)	22 499	16 247 (14 180-18 313)	19 119 (15 879-22 358)
Average total cost per patient	7858	62 521 (54 779-70 264)	22 499	27 978 (24 831-31 126)	34 543 (29 226-39 860)
Fall, any injury					
Average direct cost per patient	2318	36 776 (31 225-42 326)	6662	16 782 (14 508-19 056)	19 994 (16 237-23 751)
Average total cost per patient	2318	64 526 (55 650-73 401)	6662	28 700 (25 184-32 216)	35 826 (29 723-41 929)
Fall, minor injury					
Average direct cost per patient	2102	37 139 (31 490-42 788)	6035	17 106 (14 779-19 432)	20 034 (16 160-23 907)
Average total cost per patient	2102	65 069 (56 031-74 107)	6035	29 183 (25 582-32 784)	35 886 (29 583-42 189)
Fall, major or severe injury					
Average direct cost per patient	216	33 236 (25 652-40 820)	627	13 664 (10 800-16 527)	19 572 (14 286-24 859)
Average total cost per patient	216	59 237 (47 017-71 457)	627	24 051 (19 506-28 596)	35 186 (26 600-43 772)

**Table 4.** Cost Savings

Variable	No intervention (start of postintervention period)	Full intervention (end of postintervention period)	Difference
ITS falls per 1000 patient-days			
Rate	2.3 (2.1 to 2.4)	1.9 (1.6 to 2.1)	0.4 (0.2 to 0.6)
No injury rate	1.7 (1.5 to 1.9)	1.4 (1.1 to 1.7)	0.3 (0.1 to 0.8)
With injury rate	0.6 (0.5 to 0.7)	0.5 (0.4 to 0.6)	0.10 (0.05 to 0.14)
No. of patients (unique admissions)^a^	305 669	305 669	NA
No. of patient-days^a^	1 492 832	1 492 832	NA
No. patients			
No fall^b^	302 265 (302 026 to 302 519)	302 833 (302 564 to 303 251)	567 (284 851)
Fall, no injury^b^	2503 (2187 to 2865)	2078 (1659 to 2603)	−425 (−695 to −155)
Fall, with injury^b^	900 (806 to 1014)	758 (646 to 882)	−142 (−215 to −69)
Avgerage cost per patient, $			
No fall	24 175 (23 872 to 24 478)	24 175 (23 872 to 24 478)	NA
Fall, no injury	62 521 (54 779 to 70 264)	62 521 (54 779 to 70 264)	NA
Fall, any injury	64 526 (55 650 to 73 401)	64 526 (55 650 to 73 401)	NA
Total costs, $ (millions)			
For all patients, no fall	7307.2 (7197.6 to 7394.1)	7320.9 (7212.2 to 7414.5)	13.8 (5.9 to 21.6)
Fall, no injury	156.5 (104.8 to 230.1)	129.9 (79.5 to 209.1)	−26.6 (−45.9 to −7.3)
Fall, any injury	58.1 (39.3 to 85.1)	48.9 (31.5 to 74.1)	−9.2 (−14.6 to −3.8)
Sum of previous 3 entries	7521.8 (7229.6 to 7777.8)	7499.8 (7251.8 to 7786.5)	−22.0 (−53.8 to 10.8)
Per 1000 patient-days	5 038 626 (4 842 444 to 5 210 825)	5 023 864 (4 857 736 to 5 215 950)	−14 762 (−36 097 to 6573)

Abbreviations: ITS, interrupted time series; NA, not applicable.

^a^ Number of patients in the postintervention period.

^b^ Estimated number of patients, model-based estimate based on the ITS.

Assuming estimates of 25.5 thousand medical or surgical discharges with 123 130 000 annual patient-days nationally^19^ and extrapolating cost savings from this intervention, we project annual cost savings of $1.82 billion (direct cost savings $1.05 billion) set against projected total intervention costs of $20 million. We used the national average registered nurse hourly wage ($39.78)^20^ for this extrapolation, which results in lower intervention costs per 1000 patient-days than the weighted average hourly wage for registered nurses in Massachusetts and New York ($46.06).^21^

## Discussion

We found that the average total cost of a fall was $64 526 ($36 776 direct costs) and that the level of injury was not significantly associated with cost. The Fall TIPS Program was associated with a total cost savings of $22 million over approximately 5 years at the intervention sites, projected to a nationwide annual cost savings of $1.82 billion. Information on the cost of inpatient falls is limited, outdated, and variable,^10,11,12^ and other hospital-based fall prevention program evaluations demonstrate mixed cost-effectiveness results, in which the costs of some programs were greater than potential savings.^22^ A 2016 report^23^ contracted by the Agency for Healthcare Research and Quality estimated that the cost of a fall (any injury) was $6694 (2015 US dollars) based largely on a case-control study of an inpatient sample (62 cases [ie, patients who had a fall event]) and manual medical record review.^10^ This same report estimated that for every 1000 falls, there are 50 excess deaths. This present study used actual cost data from the EHR systems of 2 large health care systems (10 176 cases [ie, patients who had a fall event]) and determined the direct cost of a fall with any injury to be $36 776 (total cost $64 526). We stratified falls by severity, included matched controls, and aimed to provide health care leaders with information demonstrating the costs of falls and the benefits of implementing an evidence-based program. The ITS (eAppendix and eFigures 1 and 2 in Supplement 1) included a total sample of 900 635 patients and more than 74 months of data to assess how implementation of the program was associated with costs. The Fall TIPS Program saved $22.0 million in the postintervention period across 2 health care systems ($6.4 million and $15.6 million, respectively) and prevented 50 excess deaths.^23^

The costs of falls with or without injury were not appreciably different. This finding suggests that even in the absence of obvious injury, postfall evaluation and testing are extensive, and LOS is prolonged. Therefore, programs that prevent all falls provide the greatest cost-savings opportunities. To our knowledge, this is the largest study to date evaluating the cost of hospital falls, and it builds on existing literature demonstrating the cost-effectiveness of evidence-based fall prevention programs.^8,15,18^

We performed sensitivity analyses to assess variation in costs on the net benefits of the intervention. Because the material costs averaged approximately $0.88 per 1000 patient-days, we focused on uncertainty in registered nurse time. Based on a previous study, we judged the intervention to be time neutral for nurses.^18^ If instead we assume the intervention costs nurses an additional 2 minutes per shift per patient-day, then with 1.492 million patient-days in the analysis, the Fall TIPS Program increased costs by $6.88 million. This reduces the total direct cost savings per 1000 patient-days from $14 500 to $10 000 (from $8500 to $3715 for direct costs). In addition, we performed a “break-even” analysis of nurses’ time and calculated that an additional 179 registered nurse hours (including nurse champions) per 1000 patient-days could be spent on the Fall TIPS Program before the costs exceeded benefits. This is equivalent to an additional 10.75 minutes per patient each day. Thus, we conclude that the Fall TIPS Program results in net cost savings over a wide range of assumptions concerning nursing time.

In 2008, the Centers for Medicare & Medicaid Services (CMS) ended fall-related cost reimbursement,^24^ a controversial policy because some falls are not preventable.^25^ Many hospitals responded by implementing fall prevention strategies supported by little or no evidence.^26^ Today there is wide variation in the implementation of effective fall preventive strategies. Financial incentives within the national quality payment program have been used to decrease the frequency and cost of patient falls, but, to date, they address only fall injuries and a minority of cases. For example, the 2008 Inpatient Prospective Payment System initiative enacted to prevent hospital-acquired conditions (HACs) mandated that the CMS no longer pay for conditions that (1) were high cost or high volume, (2) resulted in higher payment when present as a secondary diagnosis, and (3) were considered preventable.^27^ Under the final rule, HACs are identified through claims data. Hospitals are required to report present on admission (POA) information status for principal and secondary diagnoses when submitting claims. Based on fiscal year 2011 data, 1 report found approximately 89.3 million secondary diagnosis claims, but more than 75% were reported as POA. The Falls and Trauma HAC category were the most frequently reported secondary diagnoses, but only 3.2% were coded as not POA. While only a small minority of fall injuries were coded as POA, the Falls and Trauma category contained the greatest percentage of hospital discharges (27.2%) that resulted in a reassignment of Medicare Severity–Diagnosis-Related Groups^28^ and hospitals absorbing the cost.

The CMS implemented the HAC Reduction Program^29^ to link Medicare payments to health care quality. Under that program, hospitals that rank in the worst-performing quartile receive a 1% reduction in overall Medicare payments. The total HAC score includes the CMS Patient Safety and Adverse Events Composite (CMS PSI 90), which consists of a weighted average of In-Hospital Fall with Hip Fracture Rate and 9 other HACs. Most major fall injuries, which range in severity from those that cause temporary functional impairment (ie, dislocated shoulder or broken teeth) to injuries associated with increased mortality (ie, skull fractures and subdural hematomas), are not included in this measure. Recently, the CMS announced that it plans to suppress the HAC Reduction Program payment penalties for fiscal year 2023 due to the impact of the COVID-19 public health emergency on data reporting efforts during the pandemic.^30^ Participating hospitals will not receive a total HAC score or payment penalty, but the CMS will calculate and publicly report the CMS PSI 90 scores. This pause in CMS penalty-based reductions may provide an opportunity for hospitals and health systems to reevaluate their fall prevention programs and to adopt an evidence-based program. While the CMS has implemented policy disincentives to reduce fall injuries and has created a “never event” designation for inpatient falls,^31^ it has not promoted an evidence-based fall prevention tool. Data from this study suggest that policies that incentivize hospitals to prevent all falls may be the most cost-effective. The CMS should promote evidence-based fall prevention programs like the Fall TIPS Program.

This cost-benefit analysis is based on academic medical centers and community hospitals and should be generalizable to other organizations using the Fall TIPS Program. The level of detail to which costs can be compared depends on hospital-specific cost differences. This analysis could have accounted for true one-time development costs, but other hospitals will not incur development costs because the Fall TIPS Program already exists. Conversely, costs incurred and cost benefits depend on existing organizational structures to support patient safety. Cost benefits can be extrapolated by scaling results according to the number of patients. It may be possible to use data on patient characteristics, falls or injuries, and safety culture scores to perform a more sophisticated extrapolation to typical patient populations in other types of hospitals.

This study analyzed the costs and benefits of preventing falls using the Fall TIPS Program from the health care system perspective. Findings can be used to assist other organizations in evaluating the decision to invest in implementing an evidence-based fall prevention program. Findings can also be instructive from a public policy stance as this program is beneficial for patient safety, results in cost savings, and uses validated materials that are available free of charge in 9 languages.^32^ Resources to improve patient safety are limited, and the benefits associated with the Fall TIPS Program far outweigh the associated costs.

### Limitations

We estimated total savings associated with the reduction in costs of fall-related care from the perspective of health care organizations. Data on costs were obtained through an ITS study in 2 large health care systems, and indirect costs were available in less precise forms. We did not have access to a breakdown of the components of direct and total costs; we expect that a major contributor to increased costs following a fall event is increased LOS. Our ITS design did not include a control series, so we cannot exclude confounding from co-occurring interventions or changing hospital dynamics that may have impacted hospital fall rates.

A case-control study with the same cohort was used to estimate costs based on fall injury level. To assess training costs, we multiplied the duration of training by the weighted average hourly wage for nurses by state,^21^ by the number of nurses in each health care system. While costs of direct training are calculable, the cost of time associated with the nurse champion responsibility of day-to-day training and advising fellow nurses is unknown. We included 60 minutes of general training to account for the initial 30-minute training plus 30 minutes of follow-up and reinforcement. We used $46.06 as the weighted average hourly wage for nurses. The mean hourly wage for registered nurses in the US is $39.^33^ These costs are likely lower, so cost benefits may be greater. Finally, the implementation of programs such as the Fall TIPS Program requires culture changes and coordination across multiple care teams to ensure success, the costs of which may not have been included in our estimates.

Literature on the cost of inpatient falls in the US is limited.^10,11,12^ Differences in study time frames, sample sizes, and inflation of the US dollar limit comparisons. Our analyses used 74 months of data, in which the Fall TIPS Program was implemented for 33 to 59 of these months, unit dependent. The national extrapolation is based on LOS in medical or surgical units, which cannot be adjusted to exclude intensive care unit–related LOS, which is on average longer.^34^ Finally, we did not estimate costs associated with legal liability, but reducing falls may also prevent lawsuits.

## Conclusions

Findings of this economic evaluation support that preventing falls through the use of an evidence-based program may reduce the costs associated with these adverse events. We found that the costs of falls were only marginally different by injury level. Policies that promote the reduction of all falls using evidence-based interventions may be most effective in reducing the frequency of harm and the associated costs.

## Notes

Supplement 1. **eAppendix.** Interrupted Time Series (ITS) Methods and Results **eFigure 1.** Results of the Adjusted ITS Analysis for Overall Fall Rate per 1000 Patient Days Across the Three Periods **eFigure 2.** The Results of the Adjusted ITS Analysis for Overall Fall Injury Rate per 1000 Patient Days Across the Three Periods
eReferences.
**eTable 1.** Descriptive Statistics – Site 1 (Bronx, NY) **eTable 2.** Descriptive Statistics – Site 2 (Boston, MA)

Supplement 2.
Data Sharing Statement
