# Supplementary material for: Cost of Inpatient Falls and Cost-Benefit Analysis of Implementation of an Evidence-Based Fall Prevention Program
Source: JAMA Health Forum. 2023 Jan 20;4(1):e225125. doi: 10.1001/jamahealthforum.2022.5125 (PMC9860521; doi:10.1001/jamahealthforum.2022.5125)
Supplement: Supplement 1. — eAppendix. Interrupted Time Series (ITS) Methods and Results eFigure 1. Results of the Adjusted ITS Analysis for Overall Fall Rate per 1000 Patient Days Across the Three Periods eFigure 2. The Results of the Adjusted ITS Analysis for Overall Fall Injury Rate per 1000 Patient Days Across the Three Periods eReferences. eTable 1. Descriptive Statistics – Site 1 (Bronx, NY) eTable 2. Descriptive Statistics – Site 2 (Boston, MA) [file jamahealthforum-e225125-s001.pdf]

# Supplemental Online Content

Dykes PC, Curtin-Bowen M, Lipsitz S, et al. Cost of inpatient falls and cost-benefit analysis of implementation of an evidence-based fall prevention program. *JAMA Health Forum*. 2023;4(1):e225125. doi:10.1001/jamahealthforum.2022.5125

**eAppendix.** Interrupted Time Series (ITS) Methods and Results

**eFigure 1.** Results of the Adjusted ITS Analysis for Overall Fall Rate per 1000 Patient Days Across the Three Periods

**eFigure 2.** The Results of the Adjusted ITS Analysis for Overall Fall Injury Rate per 1000 Patient Days Across the Three Periods

**eReferences.**

**eTable 1.** Descriptive Statistics – Site 1 (Bronx, NY)

**eTable 2.** Descriptive Statistics – Site 2 (Boston, MA)

This supplemental material has been provided by the authors to give readers additional information about their work.

© 2023 Dykes PC et al. *JAMA Health Forum.*

## eAppendix

**Interrupted Time Series (ITS) Methods**

*Outcomes.* Overall rate of patient falls and falls with injury per 1000 patient-days during the study period as recorded in event reporting systems.

*Statistical Methods.* The effect of the intervention on the rate of patient falls and injurious falls per 1000 patient-days was analyzed using a patient-level Poisson regression with an over-dispersion parameter fit via generalized estimating equations (GEE)[1] to account for clustering of patients within same unit. The outcome for the patient in the Poisson regression was the binary outcome fall (1=fall, 0=no fall), with the hospital length of stay (LOS) in days used as an offset term. With this model, the exponential of the Poisson regression parameters can be interpreted as rate ratios. Further, the exponential of the regression model for a subject can be interpreted as her/his fall rate per patient day. Predicted rates per 1000 patient days can be obtained by multiplying a predicted rate from the model by 1000. In the Poisson regression model, we fit segmented lines for the three periods (1. pre-intervention, 2. EHR integration/EHR go-live, and 3. post-intervention) to test for the statistical significance of observed changes in the fall rates attributable to the intervention. The length of the pre-period was the same for all units, so the model for the pre period was the same for all untis (see model below). The post period started at different points in time for the different units, and we allowed for this in the Poisson regression model, as described below. In the Poisson regression model, we adjusted for patient-level characteristics; sex, race, insurance status, age at admission, and binary Charlson Comorbidity Score (0-1; >2).[2] Predicted rates per 1,000 patient days during specific times (end of pre, beginning of post, and end of post) we obtained at the average value of these covariates.

© 2023 Dykes PC et al. *JAMA Health Forum.*

Next, we describe the Poisson regression model in more statistical detail. We let $Y_{ij}$ be the binary fall outcome (1=fall, 0=no fall) for patient $i$ in unit $j$ had a fall during their hospital stayWe let $t_{ij}$ be that patient's time of admittance relative to the start of the pre-intervention period in that unit. The patient's hospital length of stay is denoted by $LOS_{ij}$ . The fall rate per patient-day for the patient given her/his covariates $x_{ij}$ is denoted by the segmented regression model:

$$\lambda_{ij} = \exp\,[\log(LOS_{ij}) + \beta_0 + \beta_1 x_{ij} + \alpha_1 t_{ij} + \gamma_0 I(t_{ij} > \kappa_1) + \gamma_1 (t_{ij} - \kappa_1) I(t_{ij} > \kappa_{1j}) + \tau_0 I(t_{ij} > \kappa_{2j}) + \tau_1 (t_{ij} - \kappa_{2j}) I(t_{ij} > \kappa_{2j})]\ ,$$

where $I(t_{ij} > \kappa_{1j})$ equals 1 if $(t_{ij} > \kappa_{1j})$ and is equal to zero otherwise; similarly, $I(t_{ij} > \kappa_{2j})$ equals 1 if $(t_{ij} > \kappa_{2j})$ and is equal to zero otherwise.

This model allows for changes in the intercept and slope at the end of the pre period ($\kappa_1 = 15$ months) and the beginning of post periods ($\kappa_{2j}$, which differed across units from 33 to 59 months from thr start of the study). Thus, the model allows the changepoint $\kappa_{2j}$ to be different in each unit $j$. The GEE model also allows for an exchangeable correlation between patients in the same unit. Our figures are for s $\kappa_1 = 15$ months, as well as $\kappa_2$ equal to the average of the $\kappa_{2j}$'s, which is 47 months from the start of the study.We also The figures are also plotted at the average value of the $x_{ij}$'s. From this model, the rate ratios for a 1 month (consecutive month) increase in time during the pre-intervention, EHR integration/EHR go-live, and post-intervention periods are $\exp(\alpha_1)$, $\exp(\alpha_1 + \gamma_1)$, and $\exp(\alpha_1 + \gamma_1 + \tau_1)$, respectively.

In a secondary analysis to assess changes pre-to post-intervention by age (<65 years versus $\geq$65 years), we fit the adjusted Poisson regression model with an interaction between age group and period. We also assessed if the changes differed by healthcare system by fitting the

© 2023 Dykes PC et al. *JAMA Health Forum.*

adjusted Poisson regression model with an interaction between site and the time trends within period. Patient characteristics are presented as means for continuous and proportions for categorical variables. Balance in patient characteristics in the three time periods was assessed using standardized differences. All analyses used intention-to-treat principle and SAS software.[3]

**Interrupted Time Series Results**

*Interrupted Time Series Results*. In **eFigure 1**, the fall rate was close to constant in the pre-intervention period (fall rate ratio= 1.001 between consecutive months; 95% CI: 0.990-1.012; P=0.8186), significantly increased during the EHR integration/EHR go-live period (fall rate ratio= 1.003 between consecutive months; 95% CI: 1.001-1.005; P=0.0159), and significantly decreased (fall rate ratio= 0.985 between consecutive months; 95% CI: 0.978-0.993;P<0.001) in the post-intervention period from 2.27 (95% CI: 2.11, 2.44) to 1.85 (95% CI: 1.62, 2.08). The intervention was associated a 19% reduction in fall rates from the beginning to the end of post period. The downward trend in the fall rate in the post-intervention period was significantly different from the upward trend in the pre-intervention (P <0.001) and EHR integration/EHR go-live periods (P<0.001).

In the sub-analysis by age, fall rate trends within age group in the three periods were similar to the overall trends. In the post-intervention period, for age <65, the fall rate significantly decreased (P=0.020) from 2.02 (95% CI: 1.79, 2.25) to 1.76 (95% CI: 1.52, 2.01); for age ≥65, the fall rate significantly decreased (P<0.001) from 2.52 (95% CI: 2.35, 2.69) to 1.99 (95% CI: 1.67, 2.30). Fall rates decreased greatest in the ≥65 group (26.6%) versus <65 group (14.8%).

© 2023 Dykes PC et al. *JAMA Health Forum.*

In **eFigure 2**, the fall injury rate was increasing in the pre-intervention (fall injury rate ratio= 1.010 between consecutive months; 95% CI: 1.000-1.019; P=0.053) and the EHR integration/EHR go-live periods (fall injury rate ratio= 1.004 between consecutive months; 95% CI: 1.001-1.006; P=0.0155). In the post-intervention period, the fall injury rate significantly decreased (fall injury rate ratio= 0.984 between consecutive months; 95% CI: 0.977-0.992; P<0.001) from 0.67 (95% CI: 0.60, 0.74) to 0.54 (95% CI: 0.45, 0.62). The intervention was associated a 20% reduction in fall injury rates from the beginning to the end of post period. The downward trend in the fall injury rate in the post-intervention period was found to be significantly different from the upward trend in both the pre-intervention (P <0.001) and the EHR integration/EHR go-live periods (P <0.001).

In the sub-analysis by age, trends in fall injury rates within age group in the three periods were similar to the overall trends. In the post-intervention period, for age <65, the fall with injury rate significantly decreased (P=0.021) from 0.57 (95% CI: 0.48, 0.65) to 0.47 (95% CI: 0.36, 0.58); for age ≥65, the fall injury rate significantly decreased (P=0.006) from 0.68 (95% CI: 0.60, 0.76) to 0.53 (95% CI: 0.42, 0.63). The fall injury rate decrease was greatest in the age ≥65 (28.3%) versus <65 group (21.3%).

© 2023 Dykes PC et al. *JAMA Health Forum.*

**eFigure 1.** Results of the Adjusted ITS Analysis for Overall Fall Rate per 1000 Patient Days Across the Three Periods.

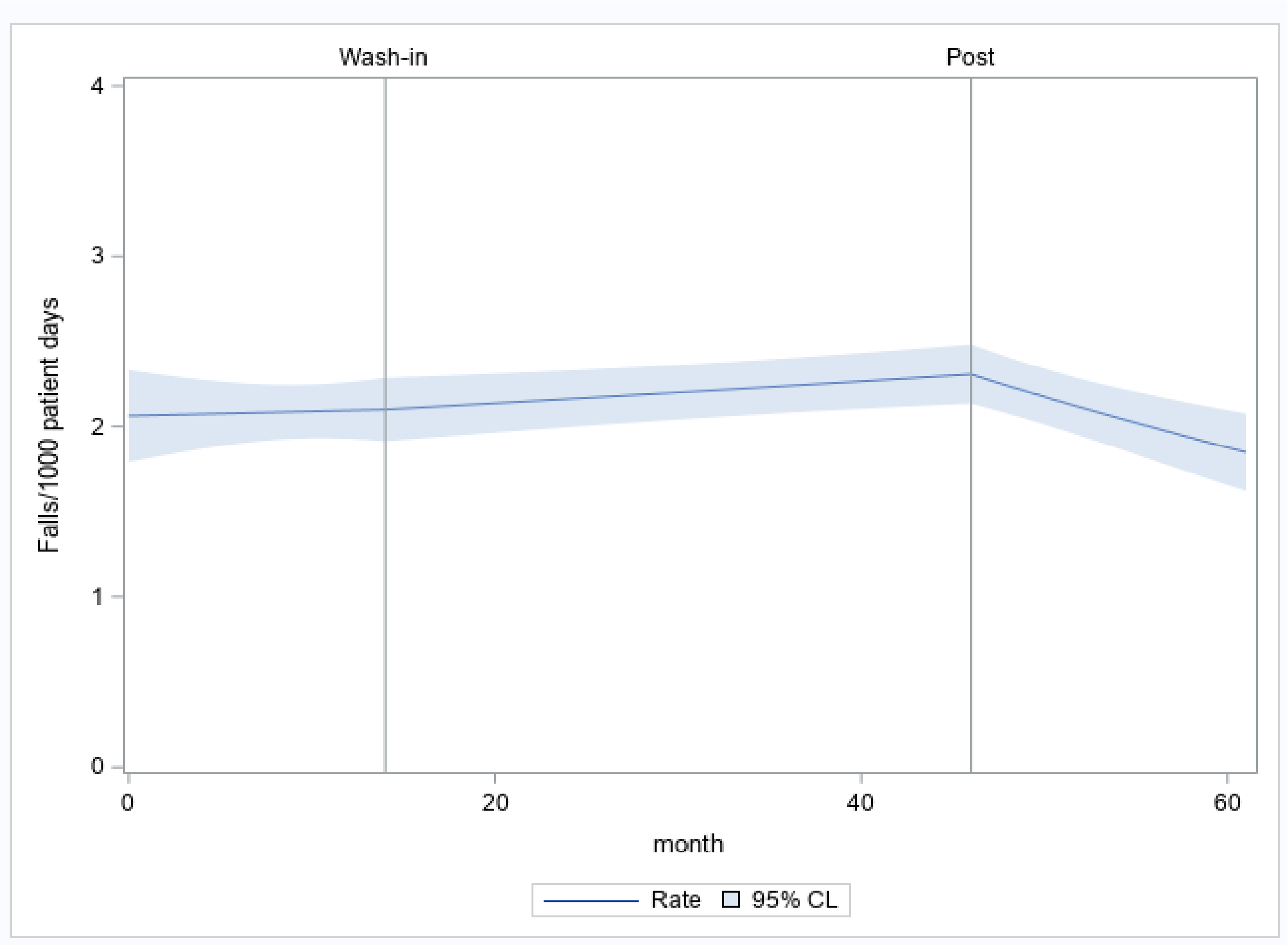


© 2023 Dykes PC et al. *JAMA Health Forum.*

**eFigure 2.** The Results of the Adjusted ITS Analysis for Overall Fall Injury Rate per 1000 Patient Days Across the Three Periods.

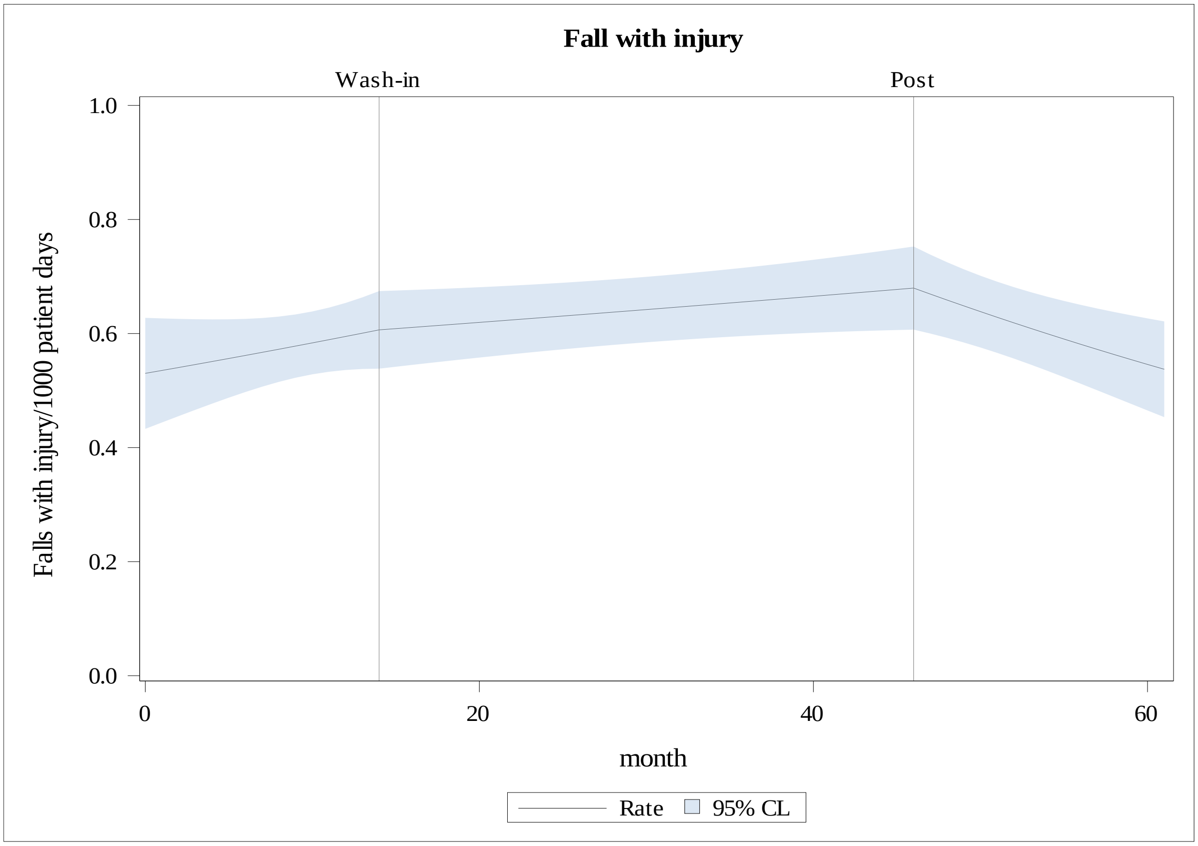


## eReferences


1. Lipsitz SR, Fitzmaurice GM, Orav EJ, Laird NM. Performance of Generalized Estimating Equations in Practical Situations. *Biometrics*. 1994;50(1):270-278. doi:10.2307/2533218

2. Charlson ME, Pompei P, Ales KL, MacKenzie CR. A new method of classifying prognostic comorbidity in longitudinal studies: Development and validation. *J Chronic Dis*. 1987;40(5):373-383. doi:10.1016/0021-9681(87)90171-8

3. SAS Institute Inc. SAS ® 9.4 Statements: Reference. Published online 2013.


© 2023 Dykes PC et al. *JAMA Health Forum.*

**eTable 1.** Descriptive Statistics – Site 1 (Bronx, NY)

| Characteristics | Before the Intervention/EHR Go-live Period, No. | EHR integration/EHR Go-live period, No. | Post Intervention, No. | Standardized Difference** | | |
|---|---|---|---|---|---|---|
| | | | | Before EHR Integration/EHR Go-live Period (%) | EHR Integration/EHR Go-live Period (%) | Post Intervention Period (%) |
| **Admissions** | 58,852 | 104,565 | 62,493 | - | - | - |
| **Patient days, No.** | 388,942.83 | 643,856.78 | 402,332.99 | - | - | - |
| **Hospital length of stay, mean (SD)** | 6.61 (10.08) | 6.16 (8.61) | 6.44 (8.91) | - | - | - |
| **Unit length of stay, mean (SD)** | 5.37 (7.51) | 5.01 (6.82) | 4.89 (6.39) | - | - | - |
| **Age, No. (%)** | | | | | | |
| <65 | 32,316 (54.91) | 56,870 (54.39) | 34,261 (54.82) | 1.05 | 0.88 | 0.17 |
| ≥65 | 26,536 (45.09) | 47,695 (45.61) | 28,232 (45.18) | -1.05 | -0.88 | -0.17 |
| **Race/ethnicity, No. (%)** | | | | | | |
| Non-white | 43,274 (79.81) | 76,931 (80.90) | 46,181 (82.62) | 2.74 | 4.45 | 7.20 |
| White | 10,949 (20.19) | 18,167 (19.10) | 9,717 (17.38) | -2.74 | -4.45 | -7.20 |
| **Ethnicity, No. (%)** | | | | | | |
| Hispanic | 22,731 (41.58) | 36,229 (39.74) | 22,849 (41.97) | 3.76 | 4.55 | 0.79 |
| Non-Hispanic | 31,933 (58.42) | 54,941 (60.26) | 31,590 (58.03) | -3.76 | -4.55 | -0.79 |
| Missing | | | | | | |
| **Sex, No. (%)** | | | | | | |
| F | 33,782 (57.40) | 59,383 (56.79) | 34,802 (55.69) | 1.23 | 2.22 | 3.45 |
| M | 25,070 (42.60) | 45,182 (43.21) | 27,691 (44.31) | -1.23 | -2.22 | -3.45 |
| **Primary Insurance, No. (%)** | | | | | | |
| Public | 48,249 (82.30) | 86,905 (83.39) | 52,436 (84.16) | 2.90 | 2.10 | 5.00 |
| Private | 10,380 (17.70) | 17,313 (16.61) | 9,867 (15.84) | -2.90 | -2.10 | -5.00 |
| **Total Charlson Comorbidity Index Score at Admission, No. (%)** | | | | | | |
| 0-1 | 25,921 (44.04) | 44,018 (42.14) | 24,542 (39.29) | 3.85 | 5.80 | 9.66 |
| ≥2 | 32,931 (55.96) | 60,440 (57.86) | 37,923 (60.71) | -3.85 | -5.80 | -9.66 |

© 2023 Dykes PC et al. *JAMA Health Forum.*

**eTable 2.** Descriptive Statistics – Site 2 (Boston, MA)

| Characteristics | Before the Intervention/ EHR Go-live Period, No. | EHR integration/EHR Go-live Period, No. | Post Intervention, No. | Standardized Difference** | | |
|---|---|---|---|---|---|---|
| | | | | Before EHR integration/EHR Go-live Period (%) | EHR Integration/EHR Go-live Period (%) | Post Intervention Period (%) |
| **Admissions** | 136,676 | 385,032 | 153,017 | - | - | - |
| **Patient days, No.** | 713,493.69 | 1,997,908.79 | 808,997.64 | - | - | - |
| **Hospital length of stay, mean (SD)** | 5.22 (6.71) | 5.19 (6.88) | 5.29 (6.88) | - | - | - |
| **Unit length of stay, mean (SD)** | 4.26 (5.31) | 4.18 (5.93) | 4.20 (5.36) | - | - | - |
| **Age, No. (%)** | | | | | | |
| <65 | 78,484 (57.42) | 202,853 (52.69) | 75,798 (49.54) | 9.54 | 6.30 | 15.86 |
| ≥65 | 58,192 (42.58) | 182,172 (47.31) | 77,219 (50.46) | -9.54 | -6.30 | -15.86 |
| **Race/ethnicity, No. (%)** | | | | | | |
| Non-white | 23,254 (17.29) | 63,757 (16.72) | 27,125 (17.90) | 1.52 | 3.11 | 1.60 |
| White | 111,238 (82.71) | 317,556 (83.28) | 124,415 (82.10) | -1.52 | -3.11 | -1.60 |
| **Ethnicity, No. (%)** | | | | | | |
| Hispanic | 8,634 (7.78) | 25,262 (7.15) | 11865 (8.16) | 2.39 | 3.79 | 1.40 |
| Non-Hispanic | 102,298 (92.22) | 327,854 (92.85) | 133,523 (91.84) | -2.39 | -3.79 | -1.40 |
| **Sex, No. (%)** | | | | | | |
| F | 70,918 (51.89) | 196,563 (51.05) | 78,604 (51.37) | 1.68 | 0.64 | 1.04 |
| M | 65,746 (48.11) | 188,456 (48.95) | 74,409 (48.63) | -1.68 | -0.64 | -1.04 |
| **Primary Insurance, No. (%)** | | | | | | |
| Public | 72,804 (54.11) | 207,009 (53.98) | 81,927 (53.65) | 0.24 | 0.67 | 0.92 |
| Private | 61,753 (45.89) | 176,452 (46.02) | 70,783 (46.35) | -0.24 | -0.67 | -0.92 |
| **Total Charlson Comorbidity Index Score at Admission, No. (%)** | | | | | | |
| 0-1 | 66,630 (50.56) | 184,203 (47.85) | 68,120 (44.52) | 5.42 | 6.69 | 12.12 |
| ≥2 | 65,156 (49.44) | 200,735 (52.15) | 84,894 (55.48) | -5.42 | -6.69 | -12.12 |

© 2023 Dykes PC et al. *JAMA Health Forum.*
