# Supplementary material for: Cost of Inpatient Falls and Cost-Benefit Analysis of Implementation of an Evidence-Based Fall Prevention Program
Source: JAMA Health Forum. 2023 Jan 20;4(1):e225125. doi: 10.1001/jamahealthforum.2022.5125 (PMC9860521; doi:10.1001/jamahealthforum.2022.5125)
Supplement: Supplement 2. — Data Sharing Statement [file jamahealthforum-e225125-s002.pdf]

Data Sharing Statement

Dykes. Cost of Inpatient Falls and Cost-Benefit Analysis of Implementation of an Evidence-based Fall Prevention Program. *JAMA Health Forum*. Published January 20, 2023. doi:10.1001/jamahealthforum.2022.5125

Data
Data available: No
